# Comparison of gamification and role-playing education on nursing students’ cardiopulmonary resuscitation self-efficacy

**DOI:** 10.1186/s12909-024-05230-7

**Published:** 2024-03-04

**Authors:** Ata Khaledi, Raziyeh Ghafouri, Sima Zohari Anboohi, Malihe Nasiri, Mohsen Ta’atizadeh

**Affiliations:** 1 Student Research Committee, School of Nursing & Midwifery, Shahid Beheshti University of Medical Sciences, Tehran, Iran; 2 Department of Medical Surgical Nursing, School of Nursing & Midwifery, Shahid Beheshti University of Medical Sciences, Vali-Asr Avenue, Cross of Vali-Asr Avenue and Hashemi Rafsanjani (Neiaiesh) Highway, Opposite to Rajaee Heart Hospital, 1996835119 Tehran, Iran; 3 Department of Basic Sciences, School of Nursing & Midwifery, Shahid Beheshti University of Medical Sciences, Tehran, Iran

**Keywords:** Cardiopulmonary resuscitation, Self-efficacy, Role playing, Education, Gamification

## Abstract

**Background:**

Cardiopulmonary resuscitation (CPR) is one of the most fundamental skills a nursing student should be trained in. Gamification in education involves using game elements to increase motivation, engagement, and personalization of the learning process. The gamification method creates competition among students using various methods, comparing to the role-playing method which is a teaching method that allows individuals to actively engage in simulated scenarios. Therefore, this research aimed to compare the effect of CPR education using gamification and role-playing on the self-efficacy of nursing students.

**Methods:**

This research was a quasi-experimental intervention type with three groups. A total of 154 nursing students participated in this study and were divided into intervention with role-playing (n = 53), gamification (n = 60) and conventional (lecture) (n = 41) groups. In the conventional method, CPR skills were taught to students using practical exercises. In the role-playing method, after training with moulages, a scenario was presented, and students were assigned roles. In the gamification method, after training with moulages, a scenario was presented, and after that, Kahoot software was used to create a sense of competition and excitement in the game. Self-efficacy scores were measured before and after interventions. Self-efficacy in CPR, knowledge, and skills of nursing students in CPR were assessed in each of the three groups using The Basic Resuscitation Skills Self- Efficacy Scale.

**Results:**

In the present study, 154 nursing students, including 92 females and 62 males, participated. There was a statistically significant difference in the mean self-efficacy scores before and after training in both the gamification and role-playing groups (P < 0.05). There was a statistically significant difference in the mean self-efficacy scores among the three groups (gamification, role-playing, and lecture) (P < 0.05).

**Conclusion:**

Based on results it can be concluded that the teaching method used in CPR training affects the self-efficacy of CPR. Active methods, have a greater impact on CPR self-efficacy.

**Supplementary Information:**

The online version contains supplementary material available at 10.1186/s12909-024-05230-7.

## Introduction

Cardiopulmonary arrest is one of the most life-threatening situations that require immediate action to protect life and prevent irreversible damage to vital body systems [1]. Cardiopulmonary resuscitation involves a series of coordinated actions by the links in the chain of survival, including rapid identification and activation, basic life support (BLS), rapid defibrillation, advanced life support, and integrated post-cardiac arrest care [2].

Inadequate CPR can ultimately lead to prolonged intervention time and poor prognosis for patients’ survival chance [2]. Cardiac arrest is responsible for 80% of hospital deaths, with survival rates of cardiac arrest patients in the hospital for 24 h and survival at discharge being 23.7% and 6.4%, respectively [3], and nurses’ preparedness for high-quality cardiac and pulmonary resuscitation improves this chance [3]. Nurses are the first responders who reach the patient’s bedside during a cardiac arrest, initiating CPR (1, 25). Effective education of nursing students [4] and ensuring the quality of nursing education [4] are vital to ensuring their role fulfillment [5, 6].

Given that traditional teaching methods have limited effectiveness in learning and education management, the use of innovative and computer-based methods can be beneficial [7]. Methods such as role play education which is a teaching method that allows individuals to actively engage in simulated scenarios to enhance learning and skill development as well as an interactive computer-based teaching method named gamification. Gamification in education involves using game elements to increase the motivation, participation, and engagement of students in the personal learning process [8]. Gamification is an active learning method that enhances learner engagement and motivation by eliciting excitement and presenting challenges. It also provides opportunities for learners to experiment and, in addition to creating challenges, allows them to experience progress and achievements [9].

Some nurses may have limited self-confidence and fear initiating CPR in patients, indicating a lack of self-efficacy in starting and performing CPR [2]. Self-efficacy in CPR is defined as the perceived ability to organize and execute the care process during resuscitation [2]. Since increased self-efficacy comes from knowledge and experience, nurses may be unprepared to perform resuscitation due to a lack of CPR training [10]. Effective CPR training can enhance self-efficacy and improve the quality of CPR performance. Role-playing training is one of the effective training methods known to impact self-efficacy, making it a suitable method for comparison with gamification in determining its effect on self-efficacy.

Identifying factors that affect the improvement of self-efficacy and psychomotor skills can help develop educational strategies to enhance these skills [11]. Considering the importance of enhancing the knowledge and skills of nursing students in CPR, the significance of the timing and method of nurse training, and the absence of studies evaluating the impact of two training methods, gamification and role-playing, on the knowledge and skills of nursing students in CPR, this study is proposed to compare the effect of gamification-based training and role-playing on the self-efficacy of nursing students at Shahid Beheshti University of Medical Sciences 2022–2023. Regarding this aim we posit that nursing students exposed to gamification-based CPR training will demonstrate a noteworthy enhancement in self-efficacy as opposed to their counterparts undergoing role-playing training.

## Methods

This research was a quasi-experimental intervention type with three groups. The study population consists of all nursing students at Shahid Beheshti University of Medical Sciences. Data was collected 30th of October 2022 to 20th August 2023.

### Participants

The participants were nursing students at Shahid Beheshti University of Medical Sciences in the years 2022 to 2023, who were selected based on entry and exit criteria. Inclusion criteria included students in the 6th semester and above who were currently taking a theoretical CPR course. Exclusion criteria included not participating in the training session (being present in the classroom for the entire session was required) and not participating in the assessment.

### Research instruments

A demographic questionnaire with 6 different questions was used to collect information regarding age, gender, academic semester, overall GPA, CPR theory course grade, and completion of an extracurricular CPR course. The Basic Resuscitation Skills Self- Efficacy Scale (BRS-SES) [12] was used to assess self-efficacy in CPR. BRS-SES consists of 18 items scored using a scale of 0–100. The items relate to recognition and alert, Automated External Defibrillator (AED) use, and the cardio-pulmonary resuscitation procedure. The BRS-SES scale has good internal consistency and reliability with an overall Cronbach’s alpha of 0.97.

The Basic Resuscitation Skills Self- Efficacy Scale [12] underwent content validity assessment after translation and back-translation of the research instrument. The quantitative and qualitative content validity was evaluated by 12 experts, including specialized nursing professors, internal medicine specialists, emergency medicine physicians, medical emergency professors, and emergency department nurses. Cronbach’s alpha was used for reliability assessment, and the obtained value of over 0.70 was considered acceptable.

### Data collection

After obtaining the necessary approvals from the Educational Deputy of the Shahid Beheshti University of Medical Sciences, as well as obtaining the ethics committee code and presenting it to the research authorities, the research team proceeded with the implementation of the study. Initially, a list of students in the 6th semester and above who met the research entry criteria was carefully prepared. This ensured that the participants had the necessary knowledge and skills to engage in the study effectively. To facilitate the research, the selected students were divided into groups of 8–10 for practical classes. Before the intervention, the average self-efficacy, knowledge, and skills of the nursing students in CPR were assessed for each of the three research groups.

This served as a baseline measurement to compare the effects of different teaching methods. In the conventional method, students were taught the skill of resuscitation through the use of moulages (simulated injuries or medical conditions) and practical exercises. This approach provided hands-on training, allowing students to practice the necessary techniques and procedures in a controlled environment. In contrast, the role-playing method which was designed by A.K incorporated an additional element of interactive learning. After receiving the conventional training with moulage, the students were presented with a scenario relevant to resuscitation. Each student was assigned a specific role within the resuscitation team, such as team leader, masseur, airway management, or medication administration, based on the scenario. The students then formed groups of four and actively participated in acting out a realistic resuscitation scene, applying their knowledge and skills in a simulated real-world context.

By implementing both the conventional and role-playing methods, the research team aimed to compare the effectiveness of these two approaches in enhancing the students’ self-efficacy, knowledge, and skills in CPR. The inclusion of role-playing added an experiential and immersive aspect to the learning process, allowing students to practice critical decision-making, teamwork, and communication skills in a dynamic setting. In the gamification method designed and prepared by R.G, after training with moulages, a scenario was presented, and students, in teams of 4, played out a resuscitation scene using the Kahoot software to create competition and engagement in the game. Self-efficacy in CPR, knowledge, and skills of nursing students in CPR were assessed in each of the three groups using The Basic Resuscitation Skills Self- Efficacy Scale [12], and the results were compared according to the research objectives.

### Data analysis

Data analysis was performed using the Statistical Package for The Social Science-20 (SPSS-20). The Kolmogorov-Smirnov test was used to check for the normality of the data. Data analysis included descriptive statistics such as mean and standard deviation, as well as inferential statistics such as Chi-square test, independent samples t-test, Wilcoxon test, and Kruskal-Wallis test. In all tests, a significance level of less than 0.05 was considered statistically significant.

## Results

In the present study, 154 nursing students (92 females and 62 males) with an average (SD) age of 23.20 (2.56) participated. Among them, 53 were trained using the role-playing method, 60 were trained using the gamification method, and 41 nursing students from the 8th semester served as the control group. The average (SD) age of students in the role-playing group was 22.64 (2.22), while in the gamification group, it was 22.25 (1.25), and for the control group, it was 23.56 (2.56). There was no significant age difference between the role-playing and gamification groups (P > 0.05). The demographic characteristics of the participants are shown in Table 1.

To assess the homogeneity of the two intervention and control groups, statistical methods were used, and the tests used are mentioned in the tables. The demographic characteristics of the participants (gender, age, semester, history of completing a CPR course, location of course completion, GPA, previous exposure to CPR) were distributed equally in the two intervention groups, gamification-based education and role-playing, and there was no statistically significant difference (P < 0.05). However, there was a statistically significant difference between the two aforementioned groups and the control group (P > 0.05).

**Table 1:** Demographic characteristics of participants in the study

Group Name Variable		Role-playing		Gamification		Control		Total	
		Frequency	Percentage	Frequency	Percentage	Frequency	Percentage	Frequency	Percentage
**Gender**	**Female**	25	16.2	34	22.1	33	21.4	92	59.7
	**Male**	28	18.2	26	16.9	8	5.2	62	40.3
**Total**		53	34.4	60	38	41	26.6	154	100
**Semester**	**6**	6	3.9	6	3.9	0	0	12	7.9
	**5**	45	29.6	54	35.5	0	0	99	65.1
	**8**	0	0	0	0	41	27	41	27
**Total**		51		60		41	27	152	100
**Gender**	**No**	49	32	36	23.5	13	8.5	99	64.1
	**Yes**	4	2.6	24	15.7	27	17.6	55	35.9
**Total**		53	34.6	60	38	41	26.6	154	100
**Place of CPR Training**	**University**	0	0	18	32.1	28	50	46	82.1
	**Red Crescent**	4	7.1	6	10.7	0	0	10	17.9
**Total**		4	7.1	24	42.8	28	50	56	36.4
**Witnessed CPR**	**No**	29	18.8	22	14.3	5	3.2	56	36.4
	**Yes**	24	15.6	38	24.7	36	23.4	98	63.6
**Total**		53	34.4	60	38	41	26.6	154	100
**Place of CPR Observation**	**Emergency**	24	24.2	34	34.4	21	21.2	79	79.8
	**Ward**	1	1	4	4	14	14	19	19.2
**Total**		25	25.2	38	38.4	35	35.3	98	100

Results of the Wilcoxon test for comparing the mean self-efficacy scores in CPR before and after training in the two intervention groups (role-playing and gamification) indicated a significant difference (P < 0.001), suggesting that both training methods were effective in improving self-efficacy. The results of the Wilcoxon test for comparing the mean self-efficacy scores in CPR after training among the three groups (gamification, role-playing, and lecture) also showed a significant difference (P < 0.05).

According to the Wilcoxon test, there was a statistically significant difference in the mean self-efficacy scores for CPR before and after education in both the gamification education and role-playing groups (P < 0.001), indicating the effectiveness of education in both groups (Table 2).

**Table 2:** Comparison of the mean self-Efficacy scores in CPR before and after education in research groups

Group Name Variable	Role-playing Group	Gamification Group	Wilcoxon Test Result
Before Intervention Mean	28.25	23.77	P < 0.001
Before Intervention Standard Deviation	15.49	6.28	
After Intervention Mean	75.49	7.45	P < 0.001
After Intervention Standard Deviation	1.78	9.49	

Based on the results of the Wilcoxon test among the three groups in the study (gamification education, role-playing, and lecture), there was a statistically significant difference in the mean scores (P < 0.05), indicating that the teaching method has an impact on self-efficacy (Table 3).

**Table 3:** Comparison of mean self-efficacy scores for CPR after education in three groups

Group Name Variable	Role-playing Group	Gamification Group	Control Group
Mean	75.49	70.45	75.39
Standard Deviation	10.78	9.94	6.93
Wilcoxon Test	P = 0.00		

## Discussion

This study aimed to compare the impact of CPR training using gamification and role-playing methods on the self-efficacy of nursing students. Demographic characteristics of participants (gender, age, academic semester, prior CPR training, place of CPR training, GPA, and CPR observation) were evenly distributed in the two intervention groups, the gamification education and role-playing group, and there were no significant differences (P > 0.05).

According to the results, there was a statistically significant difference in the mean self-efficacy scores for CPR before and after education in both the gamification education and role-playing groups (P < 0.001), indicating the effectiveness of education in both groups.

The results of the Wilcoxon test showed a statistically significant difference in the average self-efficacy scores after training between the two groups (gamification education and role-playing) and the control group (P < 0.05). Furthermore, the Wilcoxon test results revealed a statistically significant difference in the average self-efficacy scores in CPR between the three groups: the gamification education group, the role-playing group, and the 8th-semester nursing students who served as the control group (P < 0.05). Therefore, it can be concluded that the training method has an impact on self-efficacy, and the role-playing method, by providing opportunities for practice and observation of resuscitation procedures, may have a greater effect than other methods.

Sugianto and colleagues found a statistically significant difference in nurses’ knowledge before and after training using video-based and simulation-based education. However, there was no difference in the knowledge of nurses after training using video-based education and simulation-based education. They concluded that education using video-based and simulation-based methods may enhance knowledge about CPR, and there is no difference between these two methods [13].

Sun Yiyanger and colleagues also concluded in their research that teaching CPR through gamification and role-playing in neonates is effective in improving problem-solving abilities, self-confidence, and learning motivation in nursing students who participated in the experimental process. Both methods were effective [14]. The role-playing method was more effective in this study, which could be attributed to the examination context of their study. Both methods were effective in the present study in enhancing self-efficacy, but the role-playing method was more effective due to practice in simulated situations.

In a review of other studies comparing role-playing and gamification methods, no specific findings were found. Therefore, relevant studies that discussed the advantages and limitations of the role-playing method were used in this discussion. In this regard, Ahmadi and colleagues concluded based on their findings that the role-playing teaching method has an impact on the performance of nursing students in the “Principles of Higher Education to Patients” course. They recommended its use in the education of nursing students and in the training of nursing staff [15].

Asiah et al. emphasized the use of effective methods, including role-playing, along with exercises, especially in teaching critical situations [16]. Wiesefer and colleagues found that the benefits of role-playing, such as developing communication and active listening skills, fostering enthusiasm and motivation in learners, promoting collaboration, and facilitating group discussions and emotional experiences, were evident [17].

Loon Hoe et al. stated that role-playing is a useful educational method that helps adult learners quickly understand values from another person’s perspective, especially in a short period, and enhances a sense of empathy among learners [18]. Prager et al. emphasized that gamification education enhances motivation and academic performance and can be used for developing collaboration and communication skills [19].

Lee et al. also mentioned that role-playing simulations enhance decision-making and self-efficacy [20]. Role-playing is a useful educational method that helps learners quickly understand values from another person’s perspective, especially in a short period [18]. Therefore, it seems to be beneficial in teaching critical situations and rapid decision-making, such as in CPR situations.

Ong et al. highlighted the benefits of role-playing education, including understanding medical knowledge, practical application, emotional development, understanding medical terminology, improving the quality of education, gaining real-world experience by learners in a community setting, consolidating learners’ roles, successful experience of a situation, and encouraging the application of knowledge in practice [21]. Loon Hoe et al. also mentioned that role-playing is a valuable educational method for fostering empathy, allowing learners to understand values from another person’s perspective [18].

The study’s significance lies in revealing the substantial improvement in self-efficacy scores for CPR training through both gamification and role-playing methods. Notably, the results underscore the potential superiority of the role-playing approach, which, through practical engagement and observation of resuscitation procedures, appears to have a more pronounced impact on self-efficacy compared to alternative teaching methods. This approach offers additional benefits, including the transfer of empathy, collaborative learning, heightened metacognitive strategies, and tangible real-world experience, contributing to the overall enhancement of education quality and academic progress for learners.

Considering the results of the current research and the aforementioned studies, it can be stated that the teaching method of CPR has an impact on learners’ self-efficacy. The role-playing method, by providing opportunities for practice and observing resuscitation procedures, can have a greater impact than other teaching methods. Other advantages of this method include the transfer of empathy, collaborative learning, peer cooperation and assistance, the enhancement of metacognitive strategies, organization, explanation, and critical thinking, as well as gaining real-world experience in a community setting, consolidating learners’ roles, successful experience of a situation, and encouraging the practical application of knowledge. Additionally, it contributes to improving the quality of education and learners’ academic progress.

## Conclusion

Considering the significant differences among the three research groups, it can be concluded that the teaching method employed in cardiopulmonary resuscitation (CPR) affects the self-efficacy of CPR. The role-playing method, by providing opportunities for practice and observing CPR procedures, may have a more significant impact than other methods.

## Limitations

Regarding limitations in this study, study’s internal validity may be compromised by potential confounding variables that were not fully accounted for, such as individual differences in prior healthcare experiences or extracurricular CPR exposure among participants. Additionally, the absence of a placebo or sham intervention in the control group raises the possibility of participant expectations or biases influencing the observed outcomes, challenging the internal validity of the study.

### Electronic supplementary material

Below is the link to the electronic supplementary material.

Supplementary Material 1

Supplementary Material 2

## Data Availability

The datasets used and analyzed during the current study are available as supplementary file.
