# Supplementary material for: Comparison of gamification and role-playing education on nursing students’ cardiopulmonary resuscitation self-efficacy
Source: BMC Med Educ. 2024 Mar 4;24:231. doi: 10.1186/s12909-024-05230-7 (PMC10913358; doi:10.1186/s12909-024-05230-7)
Supplement: Supplementary file 2 — Supplementary Material 2 [file 12909_2024_5230_MOESM2_ESM.doc]

* Custom Tables.
CTABLES
  /VLABELS VARIABLES=group age gender semester mark course coursemark CPR CPRcourse CPRexperiance
    CPRplace q1 q2 q3 q4 q5 q6 q7 q8 q9 q10 q11 q12 q13 q14 q15 q16 q17 qq1 qq2 qq3 qq4 qq5 qq6 qq7 qq8
    qq9 qq10 qq11 qq12 qq13 qq14 qq15 qq16 qq17 selfeficacy1 selfeficacy2
    DISPLAY=LABEL
  /TABLE group [C][COUNT F40.0, TABLEPCT.COUNT PCT40.1] + age [S][MEAN, STDDEV] + gender [C][COUNT
    F40.0, TABLEPCT.COUNT PCT40.1] + semester [C][COUNT F40.0, TABLEPCT.COUNT PCT40.1] + mark [S][MEAN,
    STDDEV] + course [C][COUNT F40.0, TABLEPCT.COUNT PCT40.1] + coursemark [S][MEAN, STDDEV] + CPR
    [C][COUNT F40.0, TABLEPCT.COUNT PCT40.1] + CPRcourse [C][COUNT F40.0, TABLEPCT.COUNT PCT40.1] +
    CPRexperiance [C][COUNT F40.0, TABLEPCT.COUNT PCT40.1] + CPRplace [C][COUNT F40.0, TABLEPCT.COUNT
    PCT40.1] + q1 [C][COUNT F40.0, TABLEPCT.COUNT PCT40.1] + q2 [C][COUNT F40.0, TABLEPCT.COUNT
    PCT40.1] + q3 [C][COUNT F40.0, TABLEPCT.COUNT PCT40.1] + q4 [C][COUNT F40.0, TABLEPCT.COUNT
    PCT40.1] + q5 [C][COUNT F40.0, TABLEPCT.COUNT PCT40.1] + q6 [C][COUNT F40.0, TABLEPCT.COUNT
    PCT40.1] + q7 [C][COUNT F40.0, TABLEPCT.COUNT PCT40.1] + q8 [C][COUNT F40.0, TABLEPCT.COUNT
    PCT40.1] + q9 [C][COUNT F40.0, TABLEPCT.COUNT PCT40.1] + q10 [C][COUNT F40.0, TABLEPCT.COUNT
    PCT40.1] + q11 [C][COUNT F40.0, TABLEPCT.COUNT PCT40.1] + q12 [C][COUNT F40.0, TABLEPCT.COUNT
    PCT40.1] + q13 [C][COUNT F40.0, TABLEPCT.COUNT PCT40.1] + q14 [C][COUNT F40.0, TABLEPCT.COUNT
    PCT40.1] + q15 [C][COUNT F40.0, TABLEPCT.COUNT PCT40.1] + q16 [C][COUNT F40.0, TABLEPCT.COUNT
    PCT40.1] + q17 [C][COUNT F40.0, TABLEPCT.COUNT PCT40.1] + qq1 [C][COUNT F40.0, TABLEPCT.COUNT
    PCT40.1] + qq2 [C][COUNT F40.0, TABLEPCT.COUNT PCT40.1] + qq3 [C][COUNT F40.0, TABLEPCT.COUNT
    PCT40.1] + qq4 [C][COUNT F40.0, TABLEPCT.COUNT PCT40.1] + qq5 [C][COUNT F40.0, TABLEPCT.COUNT
    PCT40.1] + qq6 [C][COUNT F40.0, TABLEPCT.COUNT PCT40.1] + qq7 [C][COUNT F40.0, TABLEPCT.COUNT
    PCT40.1] + qq8 [C][COUNT F40.0, TABLEPCT.COUNT PCT40.1] + qq9 [C][COUNT F40.0, TABLEPCT.COUNT
    PCT40.1] + qq10 [C][COUNT F40.0, TABLEPCT.COUNT PCT40.1] + qq11 [C][COUNT F40.0, TABLEPCT.COUNT
    PCT40.1] + qq12 [C][COUNT F40.0, TABLEPCT.COUNT PCT40.1] + qq13 [C][COUNT F40.0, TABLEPCT.COUNT
    PCT40.1] + qq14 [C][COUNT F40.0, TABLEPCT.COUNT PCT40.1] + qq15 [C][COUNT F40.0, TABLEPCT.COUNT
    PCT40.1] + qq16 [C][COUNT F40.0, TABLEPCT.COUNT PCT40.1] + qq17 [C][COUNT F40.0, TABLEPCT.COUNT
    PCT40.1] + selfeficacy1 [S][MEAN, STDDEV] + selfeficacy2 [S][MEAN, STDDEV]
  /CATEGORIES VARIABLES=group gender course CPR CPRcourse CPRexperiance CPRplace q1 q2 q3 q4 q5 q6
    q7 q8 q9 q10 q11 q12 q13 q14 q15 q16 q17 qq1 qq2 qq3 qq4 qq5 qq6 qq7 qq8 qq9 qq10 qq11 qq12 qq13
    qq14 qq15 qq16 qq17 ORDER=A KEY=VALUE EMPTY=INCLUDE
  /CATEGORIES VARIABLES=semester ORDER=A KEY=VALUE EMPTY=EXCLUDE
  /CRITERIA CILEVEL=95.


Custom Tables


Notes	
Output Created	26-FEB-2024 18:36:45	
Comments		
Input	Data	D:\پایان نامه\خالدی\khaledi sub\CPR data.sav	
	Active Dataset	DataSet1	
	Filter	<none>	
	Weight	<none>	
	Split File	<none>	
	N of Rows in Working Data File	114	
Syntax	CTABLES
  /VLABELS VARIABLES=group age gender semester mark course coursemark CPR CPRcourse CPRexperiance
    CPRplace q1 q2 q3 q4 q5 q6 q7 q8 q9 q10 q11 q12 q13 q14 q15 q16 q17 qq1 qq2 qq3 qq4 qq5 qq6 qq7 qq8
    qq9 qq10 qq11 qq12 qq13 qq14 qq15 qq16 qq17 selfeficacy1 selfeficacy2
    DISPLAY=LABEL
  /TABLE group [C][COUNT F40.0, TABLEPCT.COUNT PCT40.1] + age [S][MEAN, STDDEV] + gender [C][COUNT
    F40.0, TABLEPCT.COUNT PCT40.1] + semester [C][COUNT F40.0, TABLEPCT.COUNT PCT40.1] + mark [S][MEAN,
    STDDEV] + course [C][COUNT F40.0, TABLEPCT.COUNT PCT40.1] + coursemark [S][MEAN, STDDEV] + CPR
    [C][COUNT F40.0, TABLEPCT.COUNT PCT40.1] + CPRcourse [C][COUNT F40.0, TABLEPCT.COUNT PCT40.1] +
    CPRexperiance [C][COUNT F40.0, TABLEPCT.COUNT PCT40.1] + CPRplace [C][COUNT F40.0, TABLEPCT.COUNT
    PCT40.1] + q1 [C][COUNT F40.0, TABLEPCT.COUNT PCT40.1] + q2 [C][COUNT F40.0, TABLEPCT.COUNT
    PCT40.1] + q3 [C][COUNT F40.0, TABLEPCT.COUNT PCT40.1] + q4 [C][COUNT F40.0, TABLEPCT.COUNT
    PCT40.1] + q5 [C][COUNT F40.0, TABLEPCT.COUNT PCT40.1] + q6 [C][COUNT F40.0, TABLEPCT.COUNT
    PCT40.1] + q7 [C][COUNT F40.0, TABLEPCT.COUNT PCT40.1] + q8 [C][COUNT F40.0, TABLEPCT.COUNT
    PCT40.1] + q9 [C][COUNT F40.0, TABLEPCT.COUNT PCT40.1] + q10 [C][COUNT F40.0, TABLEPCT.COUNT
    PCT40.1] + q11 [C][COUNT F40.0, TABLEPCT.COUNT PCT40.1] + q12 [C][COUNT F40.0, TABLEPCT.COUNT
    PCT40.1] + q13 [C][COUNT F40.0, TABLEPCT.COUNT PCT40.1] + q14 [C][COUNT F40.0, TABLEPCT.COUNT
    PCT40.1] + q15 [C][COUNT F40.0, TABLEPCT.COUNT PCT40.1] + q16 [C][COUNT F40.0, TABLEPCT.COUNT
    PCT40.1] + q17 [C][COUNT F40.0, TABLEPCT.COUNT PCT40.1] + qq1 [C][COUNT F40.0, TABLEPCT.COUNT
    PCT40.1] + qq2 [C][COUNT F40.0, TABLEPCT.COUNT PCT40.1] + qq3 [C][COUNT F40.0, TABLEPCT.COUNT
    PCT40.1] + qq4 [C][COUNT F40.0, TABLEPCT.COUNT PCT40.1] + qq5 [C][COUNT F40.0, TABLEPCT.COUNT
    PCT40.1] + qq6 [C][COUNT F40.0, TABLEPCT.COUNT PCT40.1] + qq7 [C][COUNT F40.0, TABLEPCT.COUNT
    PCT40.1] + qq8 [C][COUNT F40.0, TABLEPCT.COUNT PCT40.1] + qq9 [C][COUNT F40.0, TABLEPCT.COUNT
    PCT40.1] + qq10 [C][COUNT F40.0, TABLEPCT.COUNT PCT40.1] + qq11 [C][COUNT F40.0, TABLEPCT.COUNT
    PCT40.1] + qq12 [C][COUNT F40.0, TABLEPCT.COUNT PCT40.1] + qq13 [C][COUNT F40.0, TABLEPCT.COUNT
    PCT40.1] + qq14 [C][COUNT F40.0, TABLEPCT.COUNT PCT40.1] + qq15 [C][COUNT F40.0, TABLEPCT.COUNT
    PCT40.1] + qq16 [C][COUNT F40.0, TABLEPCT.COUNT PCT40.1] + qq17 [C][COUNT F40.0, TABLEPCT.COUNT
    PCT40.1] + selfeficacy1 [S][MEAN, STDDEV] + selfeficacy2 [S][MEAN, STDDEV]
  /CATEGORIES VARIABLES=group gender course CPR CPRcourse CPRexperiance CPRplace q1 q2 q3 q4 q5 q6
    q7 q8 q9 q10 q11 q12 q13 q14 q15 q16 q17 qq1 qq2 qq3 qq4 qq5 qq6 qq7 qq8 qq9 qq10 qq11 qq12 qq13
    qq14 qq15 qq16 qq17 ORDER=A KEY=VALUE EMPTY=INCLUDE
  /CATEGORIES VARIABLES=semester ORDER=A KEY=VALUE EMPTY=EXCLUDE
  /CRITERIA CILEVEL=95.	
Resources	Processor Time	00:00:00.02	
	Elapsed Time	00:00:00.03	


	Count	Table N %	Mean	Standard Deviation	
Group	role model	53	46.9%			
	gamification	60	53.1%			
	control	0	0.0%			
Age			22.30	1.79	
Gender	female	59	52.2%			
	male	54	47.8%			
academic semester	5.00	12	10.8%			
	6.00	99	89.2%			
Overall GPA			16.63	.84	
CPR theory course	No	108	95.6%			
	Yes	5	4.4%			
CPR theory course grade			16.90	.74	
Completion of an extracurricular CPR course	No	85	75.2%			
	Yes	28	24.8%			
PLace of an extracurricular CPR course	NMSBMU	18	64.3%			
	helal ahmar	10	35.7%			
	hospital	0	0.0%			
CPR experiance	No	51	45.1%			
	Yes	62	54.9%			
CPR experiance place	emergency department	58	92.1%			
	department	5	7.9%			
	critical care department	0	0.0%			
The student performs a safety assessment of the environment	not able to do	65	57.5%			
	Need help to do it	30	26.5%			
	do it with a reminder	15	13.3%			
	do with a delay less than 10 seconds	3	2.7%			
	do it perfectly	0	0.0%			
The student assesses alertness in 5 seconds	not able to do	53	46.9%			
	Need help to do it	34	30.1%			
	do it with a reminder	20	17.7%			
	do with a delay less than 10 seconds	3	2.7%			
	do it perfectly	3	2.7%			
The student calls for help out loud at the same time as the initial check and begins basic resuscitation	not able to do	56	49.6%			
	Need help to do it	16	14.2%			
	do it with a reminder	26	23.0%			
	do with a delay less than 10 seconds	15	13.3%			
	do it perfectly	0	0.0%			
The student opens the patient's airway	not able to do	61	54.0%			
	Need help to do it	28	24.8%			
	do it with a reminder	16	14.2%			
	do with a delay less than 10 seconds	2	1.8%			
	do it perfectly	6	5.3%			
The student performs the breath assessment in less than 10 seconds	not able to do	58	51.3%			
	Need help to do it	24	21.2%			
	do it with a reminder	21	18.6%			
	do with a delay less than 10 seconds	4	3.5%			
	do it perfectly	6	5.3%			
The student begins CPR without delay	not able to do	51	45.1%			
	Need help to do it	36	31.9%			
	do it with a reminder	14	12.4%			
	do with a delay less than 10 seconds	7	6.2%			
	do it perfectly	5	4.4%			
The student performs the resuscitation based on the 2020 guideline	not able to do	99	87.6%			
	Need help to do it	6	5.3%			
	do it with a reminder	8	7.1%			
	do with a delay less than 10 seconds	0	0.0%			
	do it perfectly	0	0.0%			
The student performs an effective cardiac massage with the appropriate neck	not able to do	87	77.0%			
	Need help to do it	18	15.9%			
	do it with a reminder	6	5.3%			
	do with a delay less than 10 seconds	0	0.0%			
	do it perfectly	2	1.8%			
The student breathes effectively	not able to do	85	75.2%			
	Need help to do it	16	14.2%			
	do it with a reminder	9	8.0%			
	do with a delay less than 10 seconds	1	0.9%			
	do it perfectly	2	1.8%			
The student observes the correct ratio of massage to breathing	not able to do	79	69.9%			
	Need help to do it	21	18.6%			
	do it with a reminder	11	9.7%			
	do with a delay less than 10 seconds	0	0.0%			
	do it perfectly	2	1.8%			
The student attaches the AED device to the patient as soon as possible	not able to do	81	71.7%			
	Need help to do it	19	16.8%			
	do it with a reminder	8	7.1%			
	do with a delay less than 10 seconds	2	1.8%			
	do it perfectly	3	2.7%			
The student knows how to work with the AED and the confused body performs its steps	not able to do	93	82.3%			
	Need help to do it	10	8.8%			
	do it with a reminder	7	6.2%			
	do with a delay less than 10 seconds	3	2.7%			
	do it perfectly	0	0.0%			
The student attaches the AED pads in the correct location	not able to do	97	85.8%			
	Need help to do it	5	4.4%			
	do it with a reminder	6	5.3%			
	do with a delay less than 10 seconds	0	0.0%			
	do it perfectly	5	4.4%			
During the shock, the student pays attention that no one is in contact with the patient and the bed	not able to do	91	80.5%			
	Need help to do it	12	10.6%			
	do it with a reminder	2	1.8%			
	do with a delay less than 10 seconds	3	2.7%			
	do it perfectly	5	4.4%			
The student discharges the shock correctly and quickly (by checking and announcing the shock discharge time)	not able to do	97	85.8%			
	Need help to do it	10	8.8%			
	do it with a reminder	1	0.9%			
	do with a delay less than 10 seconds	3	2.7%			
	do it perfectly	2	1.8%			
The student continues the cardiac massage without interruption	not able to do	87	77.0%			
	Need help to do it	18	15.9%			
	do it with a reminder	2	1.8%			
	do with a delay less than 10 seconds	1	0.9%			
	do it perfectly	5	4.4%			
After cardioversion shock, the student continues resuscitation quickly	not able to do	97	85.8%			
	Need help to do it	10	8.8%			
	do it with a reminder	1	0.9%			
	do with a delay less than 10 seconds	2	1.8%			
	do it perfectly	3	2.7%			
The student performs a safety assessment of the environment	not able to do	2	1.8%			
	Need help to do it	3	2.7%			
	do it with a reminder	12	10.6%			
	do with a delay less than 10 seconds	39	34.5%			
	do it perfectly	57	50.4%			
The student assesses alertness in 5 seconds	not able to do	2	1.8%			
	Need help to do it	6	5.3%			
	do it with a reminder	2	1.8%			
	do with a delay less than 10 seconds	33	29.2%			
	do it perfectly	70	61.9%			
The student calls for help out loud at the same time as the initial check and begins basic resuscitation	not able to do	1	0.9%			
	Need help to do it	2	1.8%			
	do it with a reminder	9	8.0%			
	do with a delay less than 10 seconds	20	17.7%			
	do it perfectly	81	71.7%			
The student opens the patient's airway	not able to do	1	0.9%			
	Need help to do it	1	0.9%			
	do it with a reminder	11	9.7%			
	do with a delay less than 10 seconds	29	25.7%			
	do it perfectly	71	62.8%			
The student performs the breath assessment in less than 10 seconds	not able to do	1	0.9%			
	Need help to do it	2	1.8%			
	do it with a reminder	7	6.2%			
	do with a delay less than 10 seconds	44	38.9%			
	do it perfectly	59	52.2%			
The student begins CPR without delay	not able to do	2	1.8%			
	Need help to do it	2	1.8%			
	do it with a reminder	18	15.9%			
	do with a delay less than 10 seconds	33	29.2%			
	do it perfectly	58	51.3%			
The student performs the resuscitation based on the 2020 guideline	not able to do	2	1.8%			
	Need help to do it	8	7.1%			
	do it with a reminder	15	13.3%			
	do with a delay less than 10 seconds	40	35.4%			
	do it perfectly	48	42.5%			
The student performs an effective cardiac massage with the appropriate neck	not able to do	1	0.9%			
	Need help to do it	2	1.8%			
	do it with a reminder	7	6.2%			
	do with a delay less than 10 seconds	38	33.6%			
	do it perfectly	65	57.5%			
The student breathes effectively	not able to do	1	0.9%			
	Need help to do it	3	2.7%			
	do it with a reminder	13	11.5%			
	do with a delay less than 10 seconds	26	23.0%			
	do it perfectly	70	61.9%			
The student observes the correct ratio of massage to breathing	not able to do	1	0.9%			
	Need help to do it	6	5.3%			
	do it with a reminder	7	6.2%			
	do with a delay less than 10 seconds	23	20.4%			
	do it perfectly	76	67.3%			
The student attaches the AED device to the patient as soon as possible	not able to do	1	0.9%			
	Need help to do it	12	10.6%			
	do it with a reminder	23	20.4%			
	do with a delay less than 10 seconds	43	38.1%			
	do it perfectly	34	30.1%			
The student knows how to work with the AED and the confused body performs its steps	not able to do	5	4.4%			
	Need help to do it	8	7.1%			
	do it with a reminder	24	21.2%			
	do with a delay less than 10 seconds	41	36.3%			
	do it perfectly	35	31.0%			
The student attaches the AED pads in the correct location	not able to do	2	1.8%			
	Need help to do it	0	0.0%			
	do it with a reminder	18	15.9%			
	do with a delay less than 10 seconds	34	30.1%			
	do it perfectly	59	52.2%			
During the shock, the student pays attention that no one is in contact with the patient and the bed	not able to do	2	1.8%			
	Need help to do it	2	1.8%			
	do it with a reminder	8	7.1%			
	do with a delay less than 10 seconds	55	48.7%			
	do it perfectly	46	40.7%			
The student discharges the shock correctly and quickly (by checking and announcing the shock discharge time)	not able to do	2	1.8%			
	Need help to do it	7	6.2%			
	do it with a reminder	12	10.6%			
	do with a delay less than 10 seconds	44	38.9%			
	do it perfectly	48	42.5%			
The student continues the cardiac massage without interruption	not able to do	2	1.8%			
	Need help to do it	1	0.9%			
	do it with a reminder	6	5.3%			
	do with a delay less than 10 seconds	48	42.5%			
	do it perfectly	56	49.6%			
After cardioversion shock, the student continues resuscitation quickly	not able to do	1	0.9%			
	Need help to do it	8	7.1%			
	do it with a reminder	12	10.6%			
	do with a delay less than 10 seconds	44	38.9%			
	do it perfectly	48	42.5%			
self-eficacy1			25.87	11.87	
self-eficacy2			72.81	10.60	
